# The value of non-invasive measurement of cardiac output and total peripheral resistance to categorize significant changes of intradialytic blood pressure: a prospective study

**DOI:** 10.1186/s12882-018-1087-y

**Published:** 2018-11-06

**Authors:** Yunlin Feng, Yurong Zou, Yifei Zheng, Nathan W. Levin, Li Wang

**Affiliations:** 1 Renal Division, Sichuan Academy of Medical Sciences & Sichuan Provincial People’s Hospital, No.32 West Second Section First Ring Road, Chengdu, China; 2 Mt. Sinai School of Medicine, 450 West 17th St., #842, New York, NY 10011 USA

**Keywords:** Hemodialysis, Hemodynamics, Non-invasive measurement, Cardiac output, Total peripheral resistance

## Abstract

**Background:**

Blood pressure (BP) is currently the main hemodynamic parameter used to assess the influence of fluid removal during hemodialysis session. Since BP is dependent on cardiac output (CO) and total peripheral resistance (TPRI), investigating these parameters may help to better understand the influence of fluid removal on patient’s hemodynamics. We used a novel non-invasive whole-body bio-impedance cardiography device, recently validated in hemodialysis patients, to examine mechanisms of intradialytic hemodynamics in a Chinese dialysis population.

**Methods:**

Chronic hemodialysis patients in Sichuan Provincial People’s Hospital were enrolled. Demographic data and dialysis prescriptions were collected. Hemodynamic measurements were made pre-treatment, every 20 min during treatment and immediately after treatment in each random dialysis session. These included blood pressure, cardiac index (CI), total peripheral resistance (TPRI) and cardiac power index (CPI). Patients were divided into 5 hemodynamic groups as per their major hemodynamic response to fluid removal: low CPI, low TPRI, high TPRI, High CPI and those with normal hemodynamics.

**Results:**

Twenty-seven patients were enrolled, with 12 (44.4%) males. The average age was 65 ± 12 y. The average body mass index (BMI) was 23.7 ± 3.9 kg/m2. 12 (44.4%) patients were diabetic. Three hundred twenty-four hemodynamic measurements were made. Weight, BMI, total fluid removal, pretreatment systolic BP, CI, TPRI and CI differed significantly among the 5 hemodynamic groups.11.1% of patients had low CPI, 25.9% had low TPRI, 18.5% had high CPI, 3.7% had high TPRI and 40.7% had normal hemodynamics. Hemodynamic differences among the 5 subgroups were significant.

**Conclusion:**

This technology provides multi-dimensional insight into intradialytic hemodynamic parameters, which may be more informative than blood pressure only. Using hemodynamic parameters to describe patients’ status is more specific and accurate, and could help to work out specific and effective therapeutic actions according to underlying abnormalities.

## Background

Fluid loading and removal, inherent aspects of chronic hemodialysis, are critical in patients’ care and determining outcomes. Adverse response to fluid management causes obvious symptoms, decreases quality of life, requires multiple actions of healthcare professionals and increases costs to the healthcare system. However, details of fluid management and its immediate consequence are limited by lack of information concerning intradialytic hemodynamics, largely because there are few practical methods to accurately measure the two decisive elements of blood pressure, viz. cardiac output and total peripheral resistance. Accurate measurement of cardiac output and total peripheral resistance requires the pulmonary artery catheterization determined cardiac output (CO) thermodilution technique [1], which is unrealistic in the hemodialysis clinic. Recent technological developments provide the capability to non-invasively measure hemodynamic parameters including CO during dialysis session.

These intradialytic non-invasive measurements provide opportunities to learn the full pattern of hemodynamic responses to fluid removal during hemodialysis and to intervene specifically according to deviations from normal. A novel non-invasive whole-body bio-impedance cardiography device (NICaS, NI-Medical, Israel) has been used recently to assess this in chronic hemodialysis patients [2]. The device was validated against pulmonary artery catheter thermodilution on acute heart failure patients [3, 4] and recently was also validated against Echocardiography on dialysis patients with correlation factor *r* = 0.92 which was maintained during fluid removal in maintenance hemodialysis treatments [5]. In addition, the device has been proven to be more accurate than its predecessor thoracic impedance cardiography [6].

In this study, we examined the mechanisms of intradialytic hemodynamics, including cardiac output, peripheral resistance and cardiac power, in a Chinese dialysis population, in order to evaluate different hemodynamic statuses in this population, and to provide evidence for possible specific preventive and interventional actions, particularly in the management of hypotensive episodes.

## Methods

### Subjects

Twenty-seven chronic hemodialysis patients on regular dialysis for at least 3 months in the hemodialysis center of Sichuan Provincial People’s Hospital in China were investigated. Written informed consent was obtained prior to any procedure of the study during June to September 2016. All patients received 3 maintenance hemodialysis treatment per week. The study was approved by the Institutional Review Board of the hospital and followed the ethical standards of the committee on human experimentation in our institution.

### Data collection

Demographic data including gender, age, height, weight, dialysis vintage and comorbidity of diabetes was collected. Body mass index (BMI) was calculated as weight (kg) divided by height squared (m^2^). Hemodynamic measurements in these patients were made using the device (NICaS, NI Medical, Israel), pre-treatment, every 20 min during treatment and immediately post-treatment in one random dialysis session for each individual. Blood pressure was measured at the same time as NICaS measurements. Mean arterial pressure (MAP) was calculated as (systolic blood pressure (SBP) + 2*diastolic blood pressure (DBP))/3. Dialysis prescriptions including treatment duration and total fluid removal (TFR) were recorded. Ultrafiltration rate (UFR) was expressed as fluid removal volume per kg weight per hour.

### Hemodynamic monitoring

The device utilizes the whole-body bio-impedance technology and has been fully validated to having a good correlation to pulmonary artery catheter thermodilution) [3, 4]. It is more accurate than the predecessor thoracic bio impedance technology [6] and is now US FDA approved [7]. The device measures changes in electrical resistance in the arterial system by using sensors placed on the wrist of the none-fistula hand and the contralateral ankle. These changes are converted into changes in volume of blood; stroke volume (SV) is calculated from these changes using a proprietary algorithm [3, 4]. In addition, a 1 channel electrocardiograph (ECG) is measured. Cardiac output (CO) is calculated by CO = heart rate (HR)* SV and cardiac index (CI) is calculated as dividing CO by body surface area [8]. Mean arterial pressure (MAP) is calculated from standard blood pressure. The device calculates total peripheral resistance index (TPRI = MAP/CI*80) dyn*sec/cm^5^*m^2^ [9, 10] and cardiac power index (CPI = MAP*CI/451) w/m^2^. A recent study indicated that SV measurements in NICaS are similar to and strongly correlated with echocardiographic SV measurements in a chronic hemodialysis population, confirming that the technology is a practical method for measuring SV during hemodialysis [5]. Levin et al. [2] used the device to assess hemodynamic respond to fluid removal during hemodialysis, concluding that intradialytic hypotension occurs as the result of reduction of CPI and/or TPRI and that this knowledge leads to specific actions in the management of intradialytic blood pressure.

### Patient grouping

**Fig. 1:**
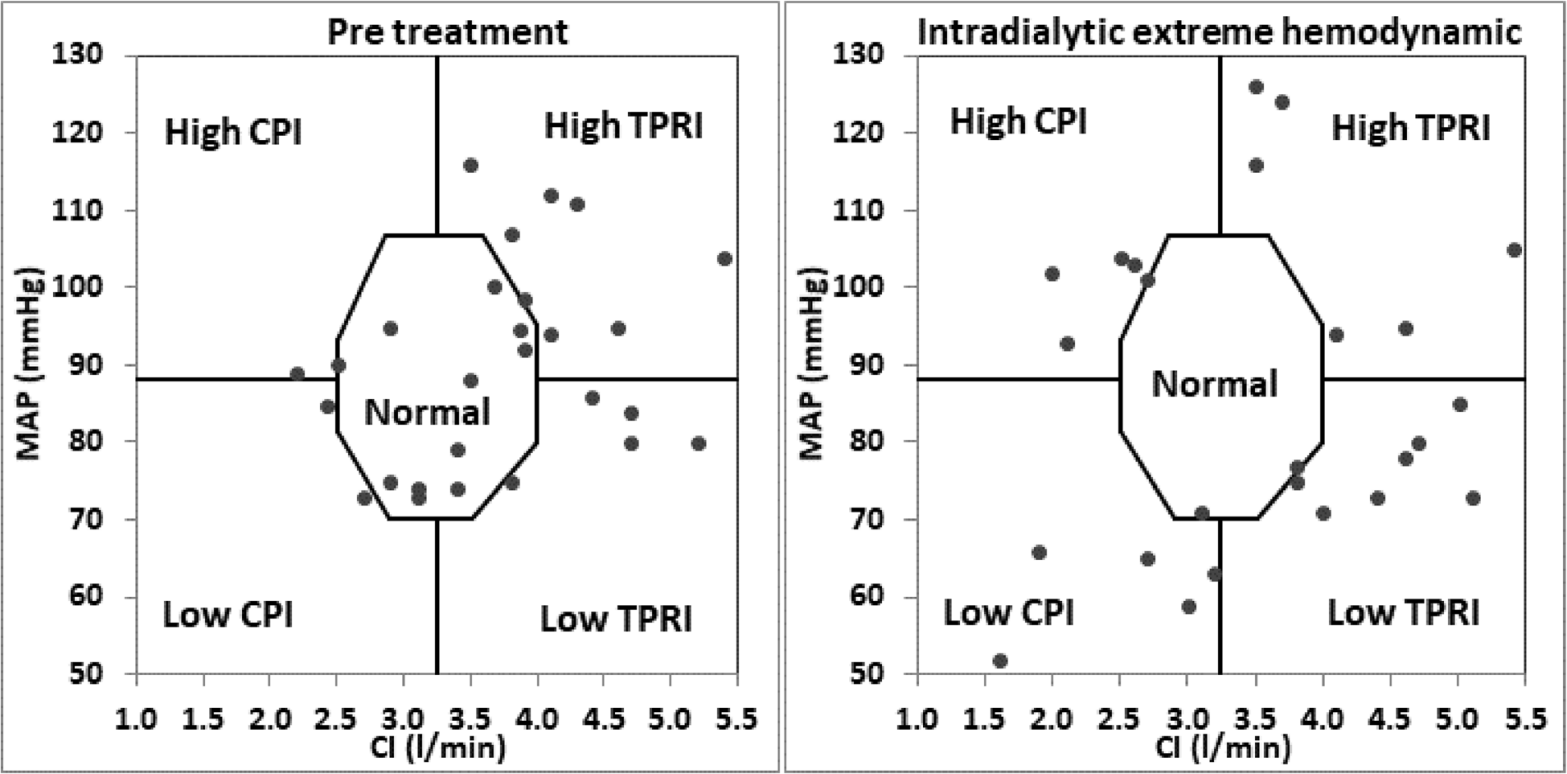
Pretreatment (on the left) and intradialytic extreme hemodynamic (on the right) hemodynamic status of all subjects. Note: Hemodynamic status of all subjects are presented using hemodynamic graphing of MAP vs CI. Normal range is illustrated in central octagon and defined as a region with BP between 70 and 105 mmHg, CI between 2.5–4.0 l/min/m^2^, CPI between 0.45–0.85 W/m^2^ and TPRI between 1600 and 3000 dyn*sec/cm*m^2^. Horizontal and vertical lines separate the 4 hemodynamic groups

Each patient was studied during one random hemodialysis treatment. Treating clinicians were blinded to the measured data. For graphical representation, a graph with CI on the x-axis and MAP on the y-axis was shown in Fig. 1. Normal hemodynamic status, as depicted in the center octagon, was defined by the normal range of MAP (70–105 mmHg), CI (2.5–4.0 l/min/m^2^), TPRI (1600–3000 dyn*Sec/cm^5^*m^2^) and CPI (0.45–0.85 W/m^2^).

After normal hemodynamics were established, patients with abnormal hemodynamics were grouped as experiencing intradialytic hypotension (IDH), defined as systolic blood pressure (SBP decreased by> 20 mmHg or MAP decreased by > 10 mmHg), and non IDH. They were further divided into 4 hemodynamic groups (in addition to normal hemodynamic) as follows:

High TPRI with MAP ≥88 mmHg and CI < 3.25 l/min/m^2^.

High CPI with MAP≥88 mmHg and CI ≥ 3.25 l/min/m^2^.

Low CPI with MAP< 88 mmHg and CI < 3.25 l/min/m^2^.

Low TPRI with MAP < 88 mmHg and CI ≥ 3.25 l/min/m^2^.

The MAP and CI measurements of each patient were plotted (see Fig. 1) in 3 measurement time points: Pretreatment measurement, maximum intradialytic hemodynamic change (defined as the maximum distance of MAP and CI from the normal range) and post treatment. These 3 points enabled graphical representation of the hemodynamic changes occurring during and after dialysis treatment in each patients.

### Statistical analysis

All statistical analyses were performed using MicroSoft Excel version 2010 (Microsoft, CA, USA). Quantitative data was expressed as mean ± standard deviation (SD). Qualitative data was expressed as number and percentage. Comparisons were made between/among groups by chi square test or ANOVA test. All tests were two-tailed. *P* < 0.05 was considered statistically significant.

## Results

**Fig. 2:**
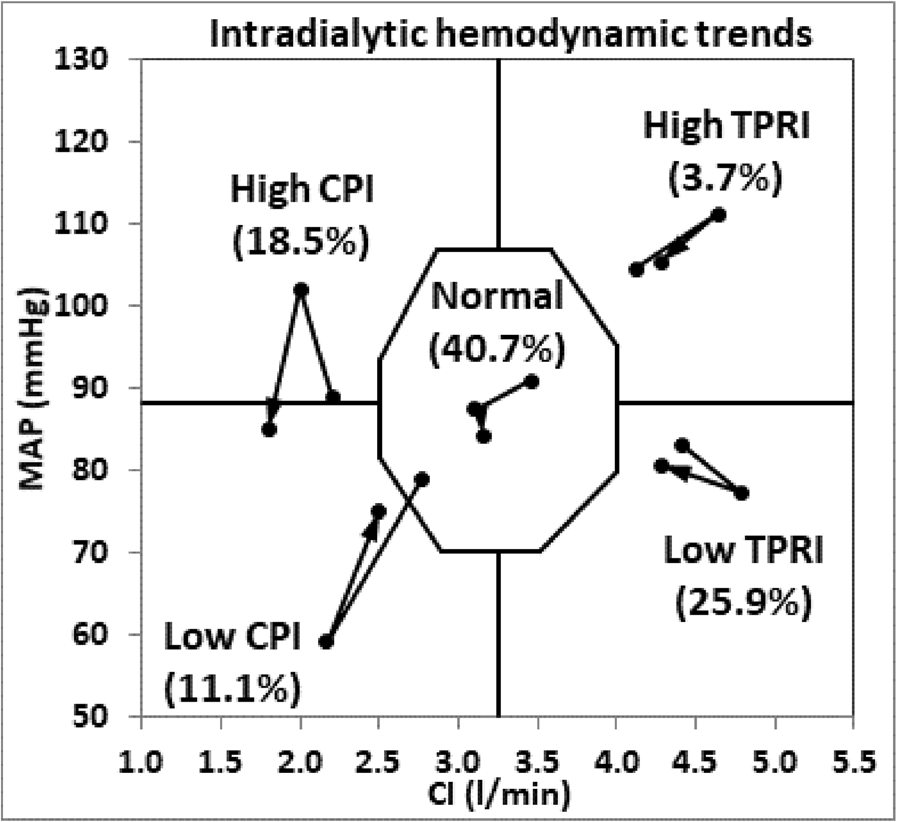
Hemodynamic changes from pretreatment to intradialytic extreme hemodynamic and to post-treatment of the 5 hemodynamic groups. Note: Hemodynamic changes from pretreatment to intradialytic extreme hemodynamic and to post-treatment (along the direction of arrows) using hemodynamic graphing of MAP vs CI. Normal range is illustrated by central octagon and defined as a region with BP between 70 and 105 mmHg, CI between 2.5–4.0 l/min/m2, CPI between 0.45–0.85 W/m2 and TPRI between 1600 and 3000 dyn*sec/cm*m2 Horizontal and vertical lines separate the 4 hemodynamic zones

The study population consisted of 27 patients, with 12 (44.4%) males. The average age was 65 ± 12 years, and the average BMI was 23.7 ± 3.9 kg/m^2^. 12 (44.4%) patients were diabetes. There were totally 324 measurements, including 27 measurements at baseline, 270 measurements made every 20 min during treatment and 27 measurements immediately after treatment. See Table 1 for demographics, fluid removal data and pretreatment hemodynamics of the IDH and non IDH groups (using above criteria). Weight and BMI were significantly higher in the IDH group than those in the non IDH group (72 ± 9 kg and 27.2 ± 4.6 kg/m^2^ vs 64 ± 12 kg and 22.6 ± 3.2 kg/m^2^, *p* = 0.015 and 0.010 respectively). Patients in the non IDH group had been on dialysis longer than those in IDH groups (104 ± 56 days vs 55 ± 16 days respectively, *p* = 0.043). Significant changes were not found in treatment duration, total fluid removal (TFR), ultrafiltration rate (UFR) and pretreatment hemodynamics between the two groups. The percentage of diabetic patients in the non IDH group (42.9%) was not significantly different from that in the IDH group (50.0%). When patients were grouped as per the 5 hemodynamic subgroups, weight and BMI were significantly different among the groups (*p* < 0.001 and *p* = 0.030 respectively), with patients in the low CPI group had the highest weight and BMI. Patients in the low CPI group and high TPRI group had significantly higher TFR (*p* = 0.010). Pretreatment SBP, CI, CPI and TPRI differed significantly among groups (*p* = 0.02, < 0.001, < 0.001 and 0.001 respectively). See Table 2 for demographics, fluid removal data and pretreatment hemodynamics. Figure 1 provided the pretreatment (on the left) and intradialytic extreme hemodynamic changes (on the right) of all subjects. Figure 2 provided the hemodynamic trends from pretreatment to intradialytic extreme hemodynamic and then to post treatment for each of the5 hemodynamic groups.

**Table 1:** Demographics, fluid removal data and pretreatment hemodynamics of the IDH and non IDH groups

Parameter	All	Non IDH	IDH	IDH vs. Non IDH
Demographics (Mean ± STD) or (n, %)				*P* value
No. of patients	27	21 (77.8%)	6 (22.2%)	
Male (n, %)	12 (44.4%)	8 (38.1%)	4 (66.7%)	0.230
Age (y)	65 ± 12	64 ± 12	72 ± 9	0.139
Weight (kg)	62 ± 12	59 ± 11	72 ± 12	0.015
BMI (kg/m^2^)	23.7 ± 3.9	22.6 ± 3.2	27.2 ± 4.6	0.010
Diabetes (n)	12 (44.4%)	9 (42.9%)	3 (50.0%)	0.072
Dialysis vintage (m)	93 ± 54	104 ± 56	55 ± 16	0.043
Fluid removal data(Mean ± STD)				*P* value
TFR [ml]	1867 ± 891	1700 ± 815	2450 ± 969	0.068
Treatment duration (hh:mm)	3:29 ± 0:28	3:28 ± 0:31	3:36 ± 0:18	0.555
UFR [ml/kg/h]	8.8 ± 3.7	8.7 ± 4.0	9.2 ± 2.3	0.750
Pretreatment Hemodynamics(Mean ± STD)				*P* value
SBP [mmHg]	136 ± 22	136 ± 22	135 ± 22	0.987
MAP [mmHg]	90 ± 13	91 ± 13	84 ± 13	0.218
CI [l/min/m^2^]	3.7 ± 0.8	3.8 ± 0.8	3.2 ± 0.7	0.115
CPI [W/m^2^]	0.74 ± 0.22	0.78 ± 0.21	0.61 ± 0.20	0.086
TPRI [dyn*sec/cm*m2]	2058 ± 503	2024 ± 519	2178 ± 465	0.519
TBW [% of body weight]	54.2 ± 8.2%	55.8 ± 8.2%	48.5 ± 6.0%	0.055

*CI* Cardiac index, *CPI* Cardiac power index, *MAP* Mean arterial pressure, *TFR* Total fluid removed, *TPRI* Total peripheral resistance index, *SBP* Systolic BP, *STD* Standard deviation, *UFR* Ultra filtration rate, *TBW* Total body water

**Table 2:** Demographics, fluid removal data and pretreatment hemodynamic data of the 5 hemodynamic groups

Para.	All	Normal	Low CPI	Low TPRI (Vasodilated)	High CPI (Hyper dynamic)	High TPRI (Vaso constricted)	*P*-value among groups
Demographics (Mean ± STD) or (n, %)							
N	27 (100%)	11 (40.7%)	3 (11.1%)	7 (25.9%)	5 (18.5%)	1 (3.7%)	
Male	12 (44.4%)	5 (45.5%)	3 (100%)	2 (28.6%)	2 (40.0%)	0 (0%)	
Age (y)	65 ± 12	67 ± 15	65 ± 2	68 ± 8	57 ± 11	73	0.497
Weight (kg)	62 ± 12	61 ± 8	81 ± 2	58 ± 12	54 ± 8	87	< 0.001
BMI (kg/m2)	23.7 ± 3.9	22.9 ± 2.6	28.3 ± 0.6	23.9 ± 5.1	20.9 ± 2.6	30.0	0.030
Diabetes	12 (44.4%)	4 (36.4%)	1 (33.3%)	4 (57.1%)	3 (60.0%)	0	0.596
Dialysis vintage (m)	93 ± 54	96 ± 42	43 ± 7	94 ± 53	121 ± 84	72	0.410
Fluid removal data (Mean ± STD)							
Duration (hh:mm)	3:29 ± 0:28	3:21 ± 0:41	3:48 ± 0:11	3:29 ± 0:11	3:39 ± 0:15	3:29	0.631
TFR (ml)	1867 ± 891	1736 ± 719	3000 ± 1082	1486 ± 511	1620 ± 782	3800	0.010
UFR (ml/kg/h)	8.8 ± 3.7	9.1 ± 4.1	9.7 ± 3.3	7.7 ± 2.9	8.4 ± 4.6	12.5	0.758
Pretreatment hemodynamic (Mean ± STD)							
SBP (mmHg)	136 ± 22	138 ± 20	132 ± 25	120 ± 13	157 ± 20	157	0.02
MAP (mmHg)	90 ± 13	91 ± 13	82 ± 9	83 ± 10	103 ± 8	89	0.58
CI (l/min/m2)	3.7 ± 0.8	3.5 ± 0.5	2.6 ± 0.3	4.4 ± 0.9	4.1 ± 0.4	2.2	< 0.001
CPI (w/m2)	0.74 ± 0.22	0.70 ± 0.16	0.47 ± 0.03	0.83 ± 0.25	0.94 ± 0.11	0.44	< 0.001
TPRI (dyn*sec/cm2*m2)	2058 ± 503	2209 ± 438	2520 ± 562	1557 ± 205	2023 ± 225	3230	0.001
TBW (%)	54.2 ± 8.2%	55.0 ± 6.4%	49.6 ± 3.6%	52.0 ± 10.0%	58.6 ± 11.3%	49	0.569

*CI* Cardiac index, *CPI* Cardiac power index, *MAP* Mean arterial pressure, *TFR* Total fluid removed, *TPRI* Total peripheral resistance, *SBP* Systolic BP, *STD* Standard deviation, *UFR* Ultra filtration rate, *TBW* Total body water

## Discussion

This study utilized a novel non-invasive regional impedance cardiography technology to illustrate the hemodynamic status of chronic hemodialysis patients during dialysis treatments with fluid removal. The results showed no significant pretreatment hemodynamic differences between IDH and non IDH patient groups. In addition, both IDH patients and non IDH patients returned to normal ranges of blood pressure, cardiac index, cardiac power index and total peripheral resistance post dialysis session. Patients were divided into hemodynamic groups as per highest and lowest intradialytic hemodynamic changes. The highest or lowest intradialytic hemodynamic measurement point in each treatment was selected as the point with the largest difference from the normal values of MAP and CI.

In healthy subjects, CI and TPRI can correctly adjust according to the change in each other, maintaining a BP in the normal range and therefore stable hemodynamics. However, in a chronic hemodialysis patient, often with impaired cardiac function, reduced vessel elasticity, or decreased autonomic function, ultrafiltration and decompensation to either decrease in CI or TPRI by various mechanisms may cause a significant BP drop during dialysis. Cardiac insufficiency may result predominantly from hypertensive myocardial, valvular disease or coronary artery disease [1]. Both systolic and diastolic dysfunctions are common in this population [11]. Hypovolemia following ultrafiltration and concomitant diastolic dysfunction may reduce preload, which results in reduction in cardiac power. Cardiac power reduction induces reduction in cardiac output or BP or a combination of both, depending upon the degree of vasoconstriction (increased TPRI). Possible reasons for decreased TPRI (vasodilatation) may include old age, diabetic angiopathy often associated with autonomic dysfunction, and atherosclerotic disease [12, 13]. In our study, the low TPRI group had highest percentage of diabetic patients (57.1%) which is frequently associated with autonomic dysfunction.

Reliance on blood pressure monitoring cannot differentiate specific mechanisms of intradialytic IDH and IH episodes. However, hemodynamic measurements can discriminate patients on the basis of their cardiac power and peripheral resistance and provide opportunities for intervention. When blood pressures routinely fall, specific prophylaxis become practical during the actual treatment. Patients with low CPI or low TPRI or both changes combined may all present with decreased BP; however, their underlying causes are different, requiring different interventions. Even in the situation when patients do not have IDH symptoms, hemodynamic analysis can indicate an abnormal change in cardiac function or in peripheral resistance as well as compensatory changes in other functions.

Reduction in cardiac power during dialysis can be as a result of reduction in preload, which may be due to hypovolemia as such, or complicated by diastolic dysfunction. Normally in this instance, the cardiac power reduction is compensated for by vasoconstriction. However, in the hemodialysis patient, the compensation is often insufficient to maintain stable hemodialysis, manifesting as a consequent BP decrease. With on-line hemodynamic monitoring regularly, decisions can be made to reduce ultrafiltration rate when a reduction in cardiac power is observed, before the blood pressure falls or to increase target weight to prevent hypovolemia. In addition, even baseline hemodynamic information, can promote specific actions targeting the underlying hemodynamic abnormality. For patients with impaired cardiac function, examinations to evaluate cardiac function, such as electrocardiograph and echocardiograph are valuable in choosing appropriate cardiotonic drugs. For patients with low TPRI (vasodilatation), dialysate temperature can be reduced or adrenergic agonists (e.g. Midodrine) can be prophylactically prescribed. Salt restriction to reduce interdialytic weight gain may also be helpful. For patients with high cardiac power (high BP and high CI), target weight could be gradually reduced. For patients with high TPRI (vasoconstriction), provision of non-dialyzable vasodilator drugs such as ARBs may be useful.

The study has several limitations. First, the sample size of 27 patients is small. However, the gender and age composition of IDH group did not differ from the non IDH group. Further studies with larger sample sizes may help to limit selection bias. Secondly, this study is cross- sectional designed. Future interventional studies designed to test IDH patients’ response to different treatments according to their hemodynamic categorization will provide useful information.

## Conclusion

This non-invasive whole-body bio-impedance cardiography technology provides insight into hemodynamic trends during dialysis. Knowledge of cardiac output index and the calculated parameters of cardiac power index and total peripheral resistance enable a multi-dimensional hemodynamic categorization, which is likely to be more informative than the current indirect dimension provided by blood pressure only. This technology may provide useful information for clinicians to make specific decisions according to underlying abnormal findings, such as increasing target weight for patients with low CPI, increasing ultrafiltration for patients with high CPI, prescribing adrenergic agonists to patients with low TPRI (vasodilatation) and vasodilators for patients with high TPRI.

## References

[1] Handt A, Farber A, Szwed J (1977). Intradialytic measurement of cardiac output by thermodilution and impedance cardiography. Clin Nephrol.

[2] Levin NW, de Abreu M, Borges LE, Tavares Filho HA, Sarwar R, Gupta S, Hafeez T, Lev S, Williams C. Hemodynamic response to fluid removal during hemodialysis: categorization of causes of intradialytic hypotension. Nephrol Dial Transplant. 2018. 10.1093/ndt/gfy048. 10.1093/ndt/gfy048 29669016

[3] Cotter G, Moshkovitz Y, Kaluski E, Cohen AJ, Miller H, Goor D, Vered Z (2004). Accurate, noninvasive continuous monitoring of cardiac output by whole-body electrical bioimpedance. Chest.

[4] Paredes OL, Shite J, Shinke T, Watanabe S, Otake H, Matsumoto D, Imuro Y, Ogasawara D, Sawada T, Yokoyama M (2006). Impedance cardiography for cardiac output estimation: reliability of wrist-to-ankle electrode configuration. Circ J.

[5] Germain Michael J., Joubert Jyovani, O'Grady Daniel, Nathanson Brian H., Chait Yossi, Levin Nathan W. (2017). Comparison of stroke volume measurements during hemodialysis using bioimpedance cardiography and echocardiography. Hemodialysis International.

[6] Cotter G, Schachner A, Sasson L (2006). Impedance cardiography revisited. Physiol Meas.

[7] N.I. MEDICAL, LTD. Bioimpedance cardiac analyzing measuring system. FDA 510k clearance No. K080941 June 18, 2009.

[8] Du Bois D, Du bois EF (1916). A formula to estimate the approximate surface area if height and weight be known. Arch Intern Med.

[9] Cotter G, Moshkovitz Y, Kaluski E (2003). The role of cardiac power and systemic vascular resistance in the pathophysiology and diagnosis of patients with acute congestive heart failure. Eur J Heart Fail.

[10] Mary E. Lough. Hemodynamic Monitoring, Evolving Technologies and Clinical Practice ELSEVIER ISBN: 978–0–323-08512-0.

[11] Wendi Bradshaw. The importance of mean arterial pressure as a patient assessment tool: in haemodialysis and acute care. Aust Nurs J. 2012;20(2):26–29. 23248911

[12] Poulin A, Bellemare PL, Fortier C (2017). Acute effects of cinacalcet on arterial stiffness and ventricular function in hemodialysis patients: A randomized double-blinded crossover study. Medicine (Baltimore).

[13] Ghigolea AB, Gherman-Caprioara M, Moldovan AR (2017). Arterial stiffness: hemodialysis versus hemodiafiltration. Clujul Med.

